# Approach to simple kidney cysts in children

**DOI:** 10.1007/s00467-024-06386-6

**Published:** 2024-04-27

**Authors:** Katherine M. Dell, Erum A. Hartung

**Affiliations:** 1 Section On Pediatric Nephrology and Hypertension, Department of Pediatrics, Cleveland Clinic Children’s, Case Western Reserve University, Cleveland, OH USA; 2 Division of Nephrology, Department of Pediatrics, Children’s Hospital of Philadelphia, Perelman School of Medicine at the University of Pennsylvania, Philadelphia, PA USA

**Keywords:** Pediatric, Kidney cyst, Polycystic kidney disease, Imaging

## Abstract

The finding of a simple kidney cyst in a child can pose a diagnostic and management challenge for pediatric nephrologists, urologists, and primary care providers. The reported prevalence varies from 0.22 to 1% in large ultrasonography-based series of more than 10,000 children each. The true prevalence, however, may be higher or lower, as factors such as variations in referral patterns, indications for ultrasonography, or technical considerations could impact prevalence rates. For many patients, simple kidney cysts may be found incidentally when imaging is performed for another indication. Although simple cysts can occur in children, they may also represent the first sign of autosomal dominant polycystic kidney disease (ADPKD) or other less common cystic kidney diseases. Definitive guidelines regarding the evaluation and monitoring of children with simple kidney cysts have not been established. The desire on the part of the practitioner and/or parents to establish a definitive diagnosis should be balanced with the cost and inconvenience of repeated imaging and visits with specialists. The goals of this review are to (1) outline the definition, epidemiology, clinical presentation, and natural history of simple kidney cysts in childhood; (2) describe clinical features that could suggest a diagnosis other than a simple kidney cyst; and (3) present a suggested framework for evaluating and monitoring of children with one or more simple kidney cysts.

**Graphical Abstract:**

**Figure:**
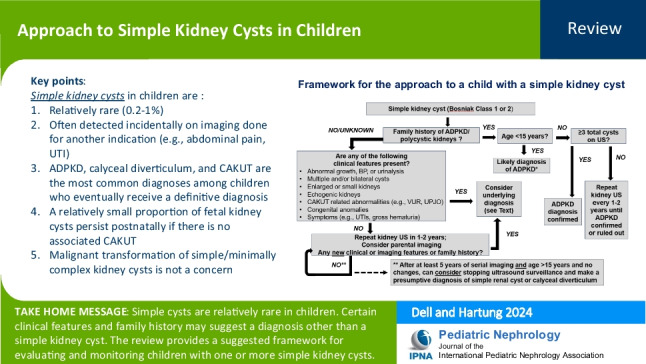
A higher resolution version of the Graphical abstract is available as Supplementary information

**Supplementary Information:**

The online version contains supplementary material available at 10.1007/s00467-024-06386-6.

## Introduction

Simple kidney cysts are being identified at increased frequency as more children undergo abdominal imaging for evaluation of symptoms such as acute abdominal pain [1]. Prenatal ultrasonography, which is now standard of care in many countries, has also led to increased identification of kidney cysts in fetuses [2, 3]. In addition, technologic innovations have resulted in increased sensitivity of such imaging, especially ultrasonography, to detect abnormalities [1]. While simple kidney cysts are common in adults and part of normal aging [4, 5], they are much less common in children [6, 7]. Importantly, a solitary simple kidney cyst can also be the first manifestation of a genetic cystic kidney disease, especially autosomal dominant polycystic kidney disease (ADPKD). Pediatric practitioners are not infrequently faced with the challenge of balancing establishing a definitive diagnosis with avoiding excessive, expensive testing and increasing parental and patient anxiety.

The goal of this review is to describe the definitions, differential diagnosis, and more common etiologies of simple kidney cysts in children and provide a proposed framework for evaluating and monitoring a child with a simple kidney cyst.

## Definitions

**Fig. 1:**
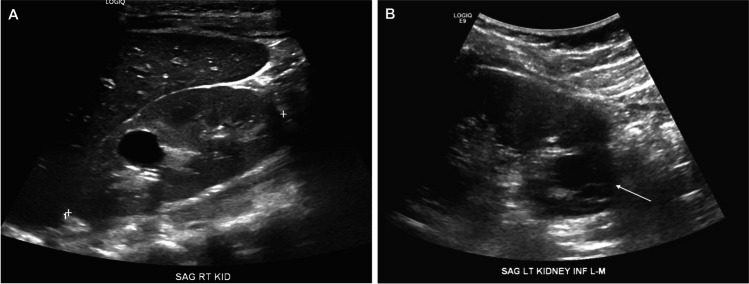
Ultrasonographic appearance of simple cysts. Kidney ultrasonography (sagittal images) illustrating: **A** a simple anechoic cyst (Bosniak Class 1) and **B** a minimally complex cyst (Bosniak Class 2) with a small septation indicated by arrow but otherwise anechoic appearance

The definition of a “simple” cyst is derived from the Bosniak classification strategy, which is an imaging-based schema developed to aid in the assessment of cystic lesions in children and adults, with a particular focus on malignancy risk [7–11]. A simple cyst (Bosniak Class 1) is a round, smooth, thin-walled (< 1 mm) cyst with homogeneous content and no septa, calcifications, echogenic foci, or solid components. There should be no enhancement on contrast computed tomography (CT) and no flow on Doppler ultrasonography. A minimally complex cyst (Bosniak Class 2) should also have smooth, thin walls but may have thin (< 1 mm) septations or fine calcifications [9, 11]. Examples of a simple cyst (Bosniak Class 1) and a minimally complex cyst (Bosniak Class 2) are shown in Fig. 1. Several studies, including those in children, have shown that Bosniak Class 1/2 cysts are not associated with malignant transformation (compared to higher Classes, 2F, 3, and 4) [7–11]. Most pediatric imaging reports typically do not specifically report Bosniak stages for Class 1/2 cysts, but such terminology may be seen occasionally.

## Epidemiology and clinical presentation of simple kidney cysts

The reported prevalence of simple kidney cysts in the pediatric populations is low, but variable. In a large series reported by McHugh et al. [6] of 16,102 children who underwent ultrasonography in the mid-late 1980s, the overall prevalence of simple kidney cysts was 0.22%. In that study, patients with abnormal kidney function, kidney dysplasia, or a known family history of polycystic kidney disease (PKD) were excluded. A more recent, even larger case series reported by Karmazyn et al. [7] of ultrasounds performed on 20,349 children during the period 2003–2011 reported a 1% prevalence of simple solitary kidney cysts. In that study, children with multiple kidney cysts or a history of a kidney tumor, surgery, transplantation, abscess, or known diagnosis of PKD, Von Hippel Lindau, or tuberous sclerosis were excluded. Patients with a known family history of cystic kidney disease, however, were not excluded. The differences in prevalence rates for these two large series likely reflects variation in exclusion criteria as well as factors such as local practice patterns in obtaining abdominal ultrasounds, patient referral base, or technical advances in ultrasonography over the last 3 decades.

Simple kidney cysts can be diagnosed in children of any age, and in most reported series, they do not show any differences in age at diagnosis [6, 7]. Presentation in fetuses has also been reported [2, 3]. In one study conducted in the 1980–1990s, the prevalence of prenatally detected simple kidney cysts by ultrasonography at 14–16 weeks gestation was 0.09% [2]. With technologic advances in fetal ultrasonography as well as a pivot to more commonly obtaining anatomical scans at 18–20 weeks, it is not known if the current prevalence is similar.

The reported distribution of females versus males among pediatric patients identified with simple kidney cysts is variable. Among the two very large cohorts discussed above, the McHugh study reported equal numbers of females and males [6], whereas the Karmazyn study found a slight female predominance (54% vs. 46%) [7]. Similar findings of no sex difference or a relatively modest female predominance was reported in two smaller studies [12, 13]. Only one study of 36 patients identified as having simple kidney cysts over a period of 11 years reported a larger female predominance (67% female vs. 36% males) [14]. Race and ethnic characteristics of the cohorts were not reported in either large series or in multiple small longitudinal follow-up series so no inferences about racial or ethnic differences can be made based on the published data.

Simple cysts in children are most commonly detected incidentally, not infrequently when imaging is performed for non-specific symptoms such as acute abdominal pain [7, 12, 13]. The cyst itself is typically asymptomatic. Urinary tract infection (UTI) is also a common indication for ultrasonography in patients found to have simple kidney cysts [6, 13, 15]. Alternatively, simple cysts may be detected as part of screening ultrasonography performed because of a family history of ADPKD, which will be discussed in more detail below.

Interestingly, several studies have noted the predominance of simple cysts in the right upper pole [6, 13, 16]. The physiologic basis for this observation is unclear. Authors of the McHugh study speculated that the difference was due to the ease of visualization of the right upper pole [6]. However, with advances in the sensitivity of ultrasonography techniques over the intervening decades, it seems unlikely that technical issues alone would explain the findings.

## Differential diagnosis

The differential diagnosis of simple kidney cysts encompasses a broad spectrum of genetic diseases, congenital/structural abnormalities, and acquired disorders. Table 1 provides a summary of the disorders that may present as simple kidney cysts in children. Disorders that present invariably with multiple kidney cysts (e.g., multicystic dysplastic kidney) are not included. Several of the more common disorders that present as simple cysts are discussed below.

**Table 1:** Differential diagnosis of simple kidney cysts in childhood

Simple kidney cyst
Polycystic kidney diseases /ciliopathies
ADPKD
ARPKD (rare)
Other ciliopathies (e.g., Juvenile nephronophthisis, Joubert syndrome) (very rare)
Tuberous sclerosis
Cystic dysplasia
Cystic dysplasia related to sporadic CAKUT
* HNF1B*-associated kidney disease
Cystic dysplasia/ “polycystic kidneys” associated with syndromic disorders
Calyceal diverticulum
Acquired cysts (history of trauma, infection, or malignancy)

## Specific diseases that may present as simple kidney cysts

### Ciliopathies

ADPKD is the most common genetic cystic kidney disease, with an incidence of approximately 1:500–1000 [17–19]. It is most commonly caused by pathogenic variants in either the *PKD1* or *PKD2* genes and is a systemic disease that may affect multiple organs including kidney, liver, heart, and vasculature [17]. ADPKD is among a heterogenous group of disorders termed *ciliopathies*, due to shared localization of the gene products on or around the primary cilia of cells, as well as important shared cellular pathogenesis (reviewed in detail in McConnachie et al. [20]). Males and females are affected equally, as are all races. The clinical course of ADPKD is highly variable and disease may not become clinically evident until the 3rd or 4th decade. Mosaicism may also contribute to the milder phenotypes [21]. Importantly, even in patients with a positive family history of ADPKD, the absence of cysts does not preclude disease until age 40 years as described in the Pei/Ravine criteria for patients aged 15 years and older [22]. Similar criteria have not been rigorously validated in younger children, but given the relatively rare occurrence of simple kidney cysts, the presence of even one cyst in a child at 50% risk of developing ADPKD is considered diagnostic by many clinicians [23]. It is also notable that 10–20% of patients have no family history of ADPKD and have a new (de novo) pathogenic gene variant [17]. The absence of a positive family history of cystic kidney disease, therefore, does not exclude the possibility of ADPKD in a child with a simple kidney cyst.

Two complementary approaches to the question of screening “at risk” children in families with ADPKD have been proposed by an international consensus conference convened in 2019 [23]. One approach is to monitor blood pressure and urinalysis yearly and perform additional evaluation if abnormal results are found, as would be done with any child. The second is to proceed directly to screening by ultrasonography. Importantly, as discussed above, the absence of a cyst in a child does not preclude the diagnosis of ADPKD since it cannot be excluded until age 40. Genetic testing may also be an option, but ultrasonography is recommended as first line for those who choose to proceed with immediate screening [23].

Autosomal recessive polycystic kidney disease (ARPKD) is a rare inherited disease, occurring at an incidence of ~ 1:20,000 patients (reviewed in [24]). It is also a ciliopathy and is caused primarily by pathogenic variants in *PKHD1*. ARPKD typically presents in the fetal or neonatal period when enlarged echogenic kidneys, reflecting diffuse microscopic dilatation of collecting tubules, are found [25]. With progressive disease, visible (macroscopic) cysts may become evident [25]. Initial presentation of ARPKD as a simple kidney cyst, however, is extremely rare but has been reported [12]. Multiple other ciliopathies are associated with kidney cysts. However, the majority are associated with obvious syndromic features (e.g., Joubert, Meckel Gruber, or Bardet-Biedl syndromes) and/or have echogenic kidneys (e.g., juvenile nephronophthisis), which can suggest the underlying diagnosis.

### Tuberous sclerosis

While not strictly classified as a ciliopathy, tuberous sclerosis (TS), an autosomal dominant disorder with variable penetrance, is also associated with kidney cysts. The protein products of the tuberous sclerosis genes, *TSC1* and *TSC2*, have significant overlap with ciliopathy pathogenesis via the mTOR pathway [26]. Approximately 50% of TS patients develop cystic kidney disease with variable degree of severity, ranging from severe bilateral disease to solitary simple kidney cysts [27]. Close attention, therefore, to any TS features (e.g., angiofibromas, Shagreen patches, developmental delays) is important in the evaluation of a child with a simple kidney cyst. Recommendations regarding the evaluation and monitoring of TS-associated kidney complications is available at https://www.tscalliance.org/healthcare-providers/.

### Cystic dysplasia

Cystic dysplasia is a group of kidney disorders with histologic features typical of kidney dysplasia, including primitive nephrons with a “fibromuscular collar,” and variable remnant elements such as bone and cartilage [28], as well as microscopic and/or macroscopic cysts. Not infrequently, the term “polycystic kidney disease” is applied to cystic dysplasia. However, it is developmentally distinct from ARPKD and ADPKD, which have normal nephron development, but then show progressively cystic tubules. Cystic dysplasia can be isolated, but most commonly occurs in the context of defined genetic syndromes (e.g., trisomies) or as a feature of congenital anomalies of the kidney and urinary tract (CAKUT), including obstructive uropathies [29]. In the past, a large majority of CAKUT cases were thought to be sporadic without a genetic cause, but more recent data show that approximately 15–20% of sporadic CAKUT is associated with copy number variants or genomic abnormalities [30, 31]. Included within the genetic forms of CAKUT/cystic dysplasia is kidney disease associated with pathogenic variants in *hepatocyte nuclear factor 1 beta (HNF1B)*, the causative gene for maturity-onset diabetes of the young, type 5 (MODY5). The phenotype of *HNF1B*-associated kidney disease is highly variable. A broad range of CAKUT disorders, including diffuse cystic dysplasia and simple kidney cysts are all reported manifestations of *HNF1B-*associated kidney disease [32].

### Calyceal diverticulum

**Fig. 2:**
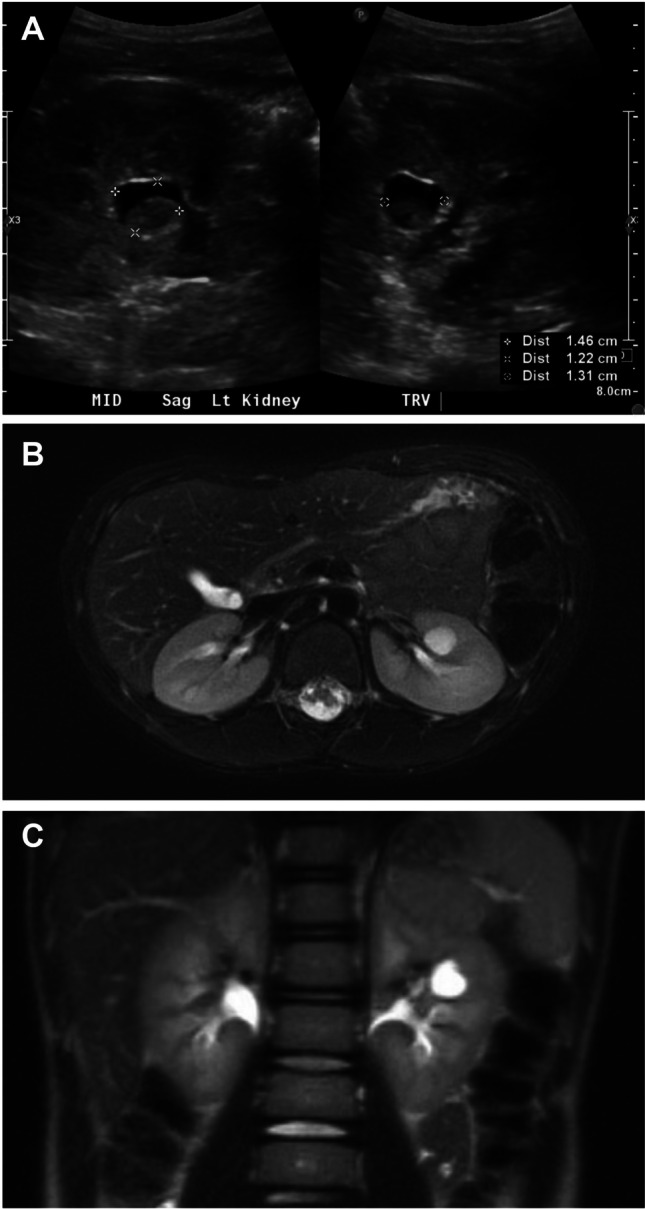
Ultrasound (US) and magnetic resonance urography (MRU) findings of calyceal diverticulum (CD). Imaging studies of a patient with a CD are shown. Ultrasound of the left kidney showing a 1.5 × 1.2 × 1.3 cm anechoic cyst containing layering echogenic debris (**A**). Axial (**B**) and coronal (**C**) T2-weighted MRU images showing a 1.3 × 1.1 × 1.3 cm T2 hyperintense lesion in the anterior interpolar region of the left kidney. The lesion abuts the collecting system and shows homogeneous hyperintensity on excretory-phase post-contrast images, consistent with filling of a CD from the urinary space

A calyceal diverticulum (CD), also called a pyelocalyceal diverticulum, is a cystic outpouching from a kidney calyx, which is connected to the calyx via an isthmus [33]. CDs not uncommonly present as simple kidney cysts on ultrasonography and in children are typically asymptomatic [15]. Symptoms, if they occur, include UTI, kidney stone, and/or flank pain, and CDs are more likely to be symptomatic than simple cysts [16]. The pathogenesis of CDs has not been clearly defined and both congenital as well as acquired etiologies (e.g., UTIs) have been proposed [16]. The overall prevalence of CD in children is poorly defined and the reported rate of CDs among pediatric patients identified with simple kidney cysts varies considerably from 0.2 to 21% [7, 15, 16]. This broad range is likely because CDs are difficult to distinguish from simple kidney cysts by ultrasonography alone. Distinguishing CDs from simple kidney cysts could be of clinical utility, as CDs are not associated with ADPKD or other systemic diseases associated with cysts. Thus, continued monitoring would not be required for those with confirmed CDs. Contrast-enhanced studies, specifically, delayed post-contrast CT [15] or magnetic resonance urography (MRU) [16] are required to demonstrate the filling of the CD, which is contiguous with the urinary space (Fig. 2). Ultrasound-based contrast-enhanced urography (CRU) has also been reported as a means to identify CDs, although the data are scarce [34]. Because of the specialized expertise, radiation exposure concerns, and/or expense associated with these imaging methods, it is likely that CDs are underdiagnosed [16].

## Natural history of simple kidney cysts in children and fetuses

Several longitudinal studies provide insights into the natural history of simple cysts in children and fetuses [2, 3, 7, 12–14, 16]. While these studies varied in number and composition of the patient population studied and duration of follow-up, they all utilized ultrasonography as the imaging modality and applied the definitions of simple or “minimally complex” cysts (i.e., Bosniak Class 1 or 2), as described above.

One of the largest studies (*n* = 87) with a median follow-up of 4 years provides important insights into the natural history of simple kidney cysts [12]. The cohort included children with ≤ 3 cysts who were diagnosed prenatally or the first year of life (*n* = 22) or after 1 year of age (*n* = 65, median [IQR] age = 7.6 [4.2, 10.6] years). Analyses were reported on the cohort as a whole and specific details about the progression of the 2 age-based subgroups were not provided. It is also notable that patients with a positive family history of cystic kidney disease were not excluded. Cysts were incidentally discovered in 39%, whereas the remaining had one or more symptoms, the most common of which was UTI (29%). Of those who eventually received a definitive diagnosis, ADPKD predominated (12.6%, *n* = 11). Of those, 4 (4.6%) had undergone screening ultrasounds because of a positive family history, but the remaining 7 were identified incidentally or because of symptoms (e.g., abdominal pain or UTI). These findings suggest that a UTI history in a patient with a simple cyst does not preclude the patient also having ADPKD. Other patients in that series who received a definitive diagnosis included 1 with ARPKD and 1 with dysplasia. A significant number (11.5%, *n* = 10) showed resolution of cysts. The remaining were diagnosed with simple kidney cyst or kidney cyst NOS. Notably, none of these patients were diagnosed with CDs, but it does not appear that any underwent MRU or delayed post-contrast CT, which would be required to diagnose CD as discussed above. No new diagnoses were made after 5 years of follow-up.

A second longitudinal study examined the outcome of 89 patients identified from a cohort of 212 patients with solitary kidney cysts, of which 204 were simple cysts. Subjects were aged ≤ 17 years and followed for at least 1 year [7]. Nine of the original 212 (4.2%) were diagnosed with CDs and were not included in the longitudinal study. Of those in the long-term simple cyst cohort, approximately 20% of cysts increased in size, 33% were stable, 6.7% were smaller, and 40% resolved. The rate of resolution was much higher than the approximately 10% reported by other studies [12, 13], for unclear reasons. The authors also noted that 4 cysts changed to complex (but not definitely malignant) and 5 patients showed an increase in number of cysts that appeared at a mean of 3.7 years (range of 1.6–6.8 years) after the first cyst appeared. This latter group could have represented subjects with ADPKD, although a definitive diagnosis was not noted.

Two studies have specifically addressed the natural history of simple kidney cysts identified during the *fetal* period. The first was conducted from 1987 to 1998 and prospectively followed 28 fetuses identified with a simple kidney cyst (without any increased echogenicity) at 14–16 weeks gestation [2]. The second was conducted in a more recent era (2005–2016) and examined simple kidney cysts identified in fetuses at 18–20 weeks gestation, which is the more typical timing for anatomy ultrasounds in fetal imaging [3]. Both studies found that a significant proportion of kidney cysts resolved postnatally, although the resolution rate of the more recent study was about half that of the older study (40% vs. 90%). Another important finding of the newer study was that over 50% of the fetuses identified as having a simple cyst were subsequently found to have a “modified” diagnosis, the majority of which fell into the category of CAKUT [3]. For both studies, only about 10% of fetal simple cysts persisted as isolated simple cysts postnatally, which were characterized as “benign” or “asymptomatic.” Neither study indicated whether those patients had a family history of kidney cysts, nor did they describe the duration of postnatal follow-up, making it difficult to confirm that those cysts were not a manifestation of early ADPKD.

In summary, these cross sectional and longitudinal studies reviewed above have important differences that make direct comparisons challenging, notably, differences in patient ages, indications for imaging, inclusion/exclusion criteria (such as positive family history of PKD), and duration of follow-up (if applicable). Despite these differences, in aggregate, they provide important insights and the following themes emerge regarding simple/minimally complex kidney cysts *in children*:

1. They are relatively rare (0.2–1%).
2. They are often detected incidentally when imaging is done for another indication (e.g., abdominal pain, UTI).
3. ADPKD, calyceal diverticulum, and CAKUT are the most common diagnoses among those who receive an eventual definitive diagnosis.
4. If they are identified in the fetus, and not found to be associated with CAKUT, a relatively small number show persistence postnatally over time.
5. Malignant transformation of simple/minimally complex renal cysts is not a concern.

## Evaluation and management of a child with a simple kidney cyst

There are currently no formal published consensus guidelines for the evaluation and management of a simple kidney cyst in a child. A number of clinical factors should be considered in the initial evaluation that could suggest a diagnosis other than simple cyst. These include the presence of multiple or bilateral cysts, a positive family history of cysts, the presence of other clinical features, such as congenital anomalies, other structural urogenital findings, or hypertension, and/or symptoms, such as flank pain. It is important to note that 10–15% of patients with ADPKD do not have a positive family history (and are considered to have new pathogenic variants), so the absence of a positive family history does not preclude disease.

**Fig. 3:**
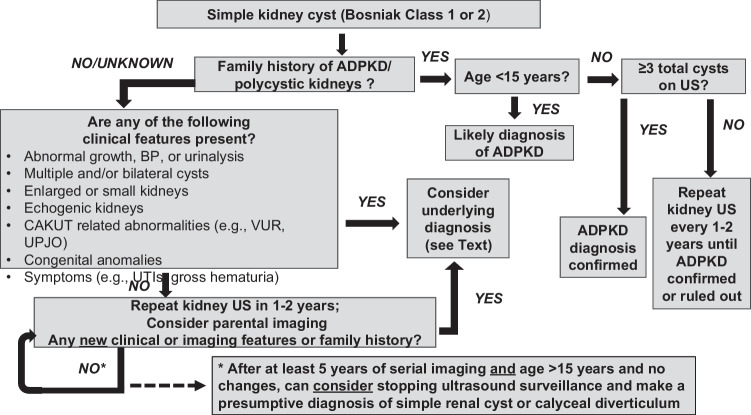
Framework for the approach to a child with a simple kidney cyst. A proposed framework for evaluation and management of a child with a simple kidney cyst is shown. ADPKD, autosomal dominant polycystic kidney disease; VUR, vesico-ureteral reflux; UTI, urinary tract infection; UPJO, ureteral pelvic junction obstruction; CAKUT, congenital anomalies of the kidney and urinary tract

The approach to serial imaging varies among pediatric nephrologists as well as between pediatric nephrologists and pediatric urologists. In a survey study addressing this question, pediatric urologists (*n* = 128) generally recommended more frequent follow-up (every 6–12 months), whereas pediatric nephrologists (*n* = 37) recommended follow-up every 1–2 years. Pediatric nephrologists were also likely to increase frequency of imaging in patients who developed bilateral cysts or chronic kidney disease [35]. Considering the existing published data (with themes summarized above) as well as our collective clinical experience, we propose a framework to approach the evaluation and monitoring of a child with a simple kidney cyst (Fig. 3). A key driver in this algorithm is the recognition that early ADPKD is indistinguishable from a simple kidney cyst and that “ruling out” or “ruling in” ADPKD may require serial imaging into adolescence. In addition, it should be noted that this is not a “one size fits all” approach and the child’s specific clinical features, age, parental anxiety, and the availability of resources should be considered in terms of providing a personalized approach. For instance, MRU can distinguish a CD from a simple kidney cyst but is costly and requires specialized expertise, equipment, and sedation in younger children. In an older child who is cared for at an institution with the requisite equipment and expertise, for whom cost would not be a major barrier, an MRU could be considered at the initial evaluation. However, in a younger child, an MRU might only be considered if there are obvious symptoms or if the cyst is very large. In other instances, including resource-limited areas, serial ultrasonography at the suggested intervals (if available) is a very reasonable approach.

Taking these factors into account, the first consideration is the family history of ADPKD/polycystic kidneys. Since patients with an affected ADPKD parent have a 50% risk of having ADPKD, the likelihood that a simple cyst is the manifestation of early disease is much higher for these children than the general population. In fact, many investigators consider even a single cyst in an “at risk” child under 15 years of age to be highly suggestive of ADPKD [17]. It is possible, however, that the parent may have ADPKD and be unaware of the diagnosis. Assessing parental history of hypertension in early adulthood or a family history of early stroke (e.g., from cerebral aneurysm) is an important additional component of the family history. Age, in the presence of a positive ADPKD family history, is also a key feature, as ADPKD can be diagnosed (but not ruled out) as early as 15 years of age utilizing the Pei-Ravine criteria [36].

The next step is assessment of the child’s history and clinical features. Physical exam findings such as elevated blood pressure or syndromic features suggest diagnoses other than simple kidney cysts. A history of UTI could suggest CAKUT. Abnormal urinalysis (e.g., proteinuria) also suggests alternative diagnoses. Laboratory findings of abnormal serum creatinine or metabolic acidosis suggest chronic kidney disease, which is not a feature of simple cysts. Imaging findings suggestive of underlying kidney disease (e.g., echogenic kidneys) and/or urologic abnormalities (e.g. hydronephrosis or bladder wall thickening) are also informative.

In the absence of evidence for an underlying kidney disease, we recommend serial ultrasonography every 1–2 years and yearly blood pressure monitoring. It is generally not recommended that a child with a single simple kidney cyst undergo genetic testing [37]. Pediatric nephrology follow-up can be at a less frequent interval (e.g., every 2–3 years), if the imaging findings are stable and no hypertension is identified. In addition, if the parents have not had kidney imaging performed after the age of 30 years (when most cases of ADPKD are evident), ultrasonography can be considered (and could be obtained by the parent’s primary care provider). Finally, because it may take several years for overt findings of ADPKD to be present, it is suggested that imaging continues for at least 5 years and/or until the child is well into adolescence. Since the Pei-Ravine criteria for diagnosis of ADPKD in a patient with an affected parent can be invoked as early as age 15 years, imaging until that point is a reasonable approach.

## Conclusions

Simple kidney cysts in children are relatively rare but are being identified at increasing frequency as children more frequently undergo diagnostic abdominal imaging and radiographic techniques have improved sensitivity. Not uncommonly, simple cysts are identified incidentally during imaging for another indication. There are currently no formal published consensus guidelines for the evaluation and management of simple kidney cysts in children. Clinical features and family history, however, can suggest alternative diagnoses, such as ADPKD, cystic dysplasia, or CD, which may inform additional evaluation. Serial ultrasonography is typically performed in children with a solitary simple cyst although the optimal frequency has not been established. We present a suggested framework for approaching the evaluation and monitoring of a child with a simple kidney cyst, while also emphasizing the need for a personalized, tailored approach.

## Supplementary Information

Below is the link to the electronic supplementary material.

Graphical abstract (PPTX 196 KB)
