# Supplementary material for: Approach to simple kidney cysts in children
Source: Pediatr Nephrol. 2024 Apr 27;39(12):3387–95. doi: 10.1007/s00467-024-06386-6 (PMC11511774; doi:10.1007/s00467-024-06386-6)
Supplement: Supplementary file 1 — Graphical abstract (PPTX 196 KB) [file 467_2024_6386_MOESM1_ESM.pptx]

## Slide 1
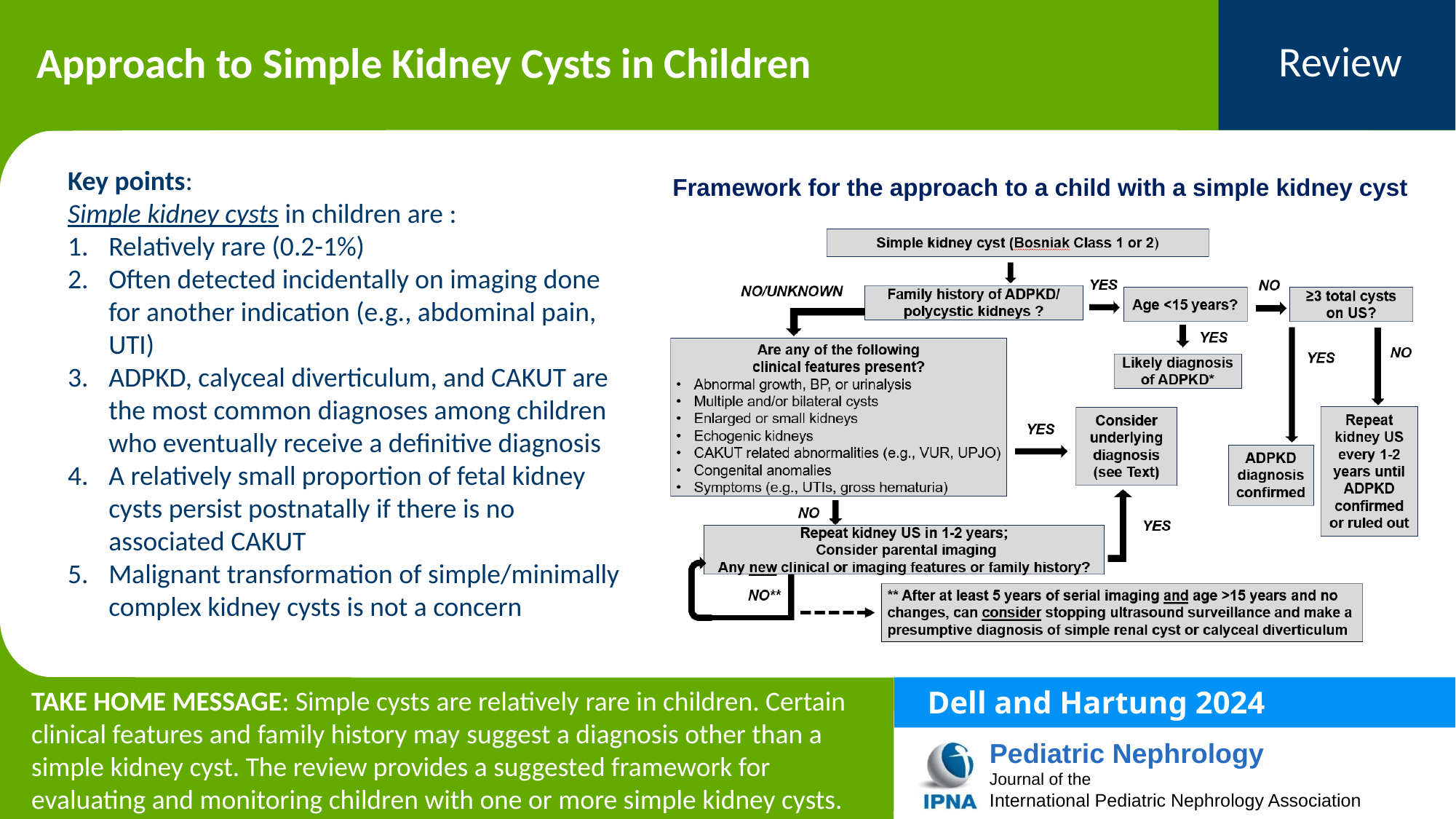

Approach to Simple Kidney Cysts in Children
Key points:
Simple kidney cysts in children are :
Relatively rare (0.2-1%)
Often detected incidentally on imaging done for another indication (e.g., abdominal pain, UTI)
ADPKD, calyceal diverticulum, and CAKUT are the most common diagnoses among children who eventually receive a definitive diagnosis
A relatively small proportion of fetal kidney cysts persist postnatally if there is no associated CAKUT
Malignant transformation of simple/minimally complex kidney cysts is not a concern
Framework for the approach to a child with a simple kidney cyst
TAKE HOME MESSAGE: Simple cysts are relatively rare in children. Certain clinical features and family history may suggest a diagnosis other than a simple kidney cyst. The review provides a suggested framework for evaluating and monitoring children with one or more simple kidney cysts.
Dell and Hartung 2024
